# Compact Harmonic Vernier Sensor Based on an In-Fiber FPI with Three Reflector System for Simultaneous Gas Pressure and Temperature Measurement

**DOI:** 10.3390/s23084142

**Published:** 2023-04-20

**Authors:** Jinxiao Dan, Wenjie Dang, Zeren Li, Pengyu Nan, Guoguo Xin, Kok-Sing Lim, Harith Ahmad, Hangzhou Yang

**Affiliations:** 1 School of Physics, Northwest University, Xi’an 710069, China; danjxiao@163.com (J.D.);; 2 Photonics Research Centre, University Malaya, Kuala Lumpur 50603, Malaysia

**Keywords:** optical fiber sensor, Vernier effect, gas pressure and temperature, Fabry–Perot interferometer

## Abstract

In this work, we proposed a sensitivity-enhanced temperature sensor, a compact harmonic Vernier sensor based on an in-fiber Fabry–Perot Interferometer (FPI), with three reflective interfaces for the measurement of gas temperature and pressure. FPI consists of air and silica cavities formulated by single-mode optical fiber (SMF) and several short hollow core fiber segments. One of the cavity lengths is deliberately made larger to excite several harmonics of the Vernier effect that have different sensitivity magnifications to the gas pressure and temperature. The spectral curve could be demodulated using a digital bandpass filter to extract the interference spectrum according to the spatial frequencies of resonance cavities. The findings indicate that the material and structural properties of the resonance cavities have an impact on the respective temperature sensitivity and pressure sensitivity. The measured pressure sensitivity and temperature sensitivity of the proposed sensor are 114 nm/MPa and 176 pm/°C, respectively. Therefore, the proposed sensor combines ease of fabrication and high sensitivity, making it great potential for practical sensing measurements.

## 1. Introduction

As an important physical parameter, gas pressure plays a great role in many research fields. Compared to traditional electrical pressure sensors, fiber optic pressure sensors have the advantages of compact size, light weight, small shape, anti-electromagnetic interference, high accuracy, and high sensitivity [1,2,3,4,5]. Optical fiber sensors for gas pressure measurement have widespread applications in the fields of aerospace [6,7,8], meteorological monitoring [9], civil engineering [10,11], and other industrial fields. One of the widely used optical fiber gas pressure sensors currently is based on Fabry–Perot interferometer (FPI) [12,13,14,15]. The mechanism of the FPI-based sensor in the pressure measurement is associated with the changes in the internal refractive index (RI) [15,16] and the cavity length of the interferometer [12,17]. Several methods for measuring gas pressure based on FPI have been proposed. For example, Chen et al. [18] designed an FPI sensor based on core-offset fusion with a pressure sensitivity of 4.314 nm/MPa within the 0.4–1.0 MPa. Liang et al. [19] proposed a diaphragm-free FPI sensor with a pressure sensitivity of 4.28 nm/MPa in the range of 0 to 0.7 MPa and temperature sensitivity of 14.8 pm/°C within 20 to 800 °C. However, the sensitivities of the aforementioned sensors are insufficient for the actual applications and practice.

In order to enhance the sensitivity and increase the resolution of the measurements, researchers have been applying the Vernier effect to FPI-based optical fiber sensors [20,21]. The optical Vernier effect has become a reliable and effective method to improve the accuracy of the sensor measurement [22,23]. Generally, Vernier-based sensors are composed of two parallel or cascaded interferometers with a small difference in free spectral range (FSR), which is deliberately done to attain an interference spectrum with a sensitive envelope to the parameter of interest. Despite the advantages of high accuracy and good resolution, the fabrication process for Vernier-based sensors is sophisticated and challenging, notably due to the difficulty in the precision control of the cavity length, which is an important parameter to manipulate the FSRs of the interferometers and the sensitivity of the envelope. To address this problem, Gomes et al. proposed the use of the harmonic Vernier effect [24], which exploits the higher-order harmonics in the interference spectrum that offer better flexibility in the design and fabrication of the interferometer. Contrary to the conventional Vernier effect, the difference in FSRs of the two interferometers is deliberately made larger to excite more harmonics in the interference spectrum. The magnification factor of harmonics is proportional to its order. Gomes et al. [25] first combined hollow microspheres with an optical sensitivity magnification, through harmonics of the Vernier effect, achieving a strain sensitivity of 146.3 pm/µε, which is 120 times higher than that of a fiber Bragg grating, with an amplification factor of 23, and the silica cavities have high-temperature sensitivity and are barely responsive to strain temperature sensitivity of 90 pm/°C; however, the structure of the sensor is complex. Applying the harmonic Vernier effect, Yang et al. [26] fabricated a Fabry–Perot (FP) cavity by incorporating 2 silicon capillary segments into a fiber structure with a pressure sensitivity of 80.8 nm/MPa and a temperature sensitivity of 179.99 pm/°C. From these studies, it can be found that the application of the harmonic Vernier effect can significantly improve the sensitivity of sensors and reduce the difficulty of sensor fabrication [26,27,28].

In this work, a high-sensitive harmonic Vernier sensor based on an in-fiber FPI sensor is experimentally demonstrated for the measurement of gas temperature and pressure. This report provides the detailed fabrication steps and parameters to produce an FPI with three reflective interfaces. The sensor introduces optical harmonics into the Vernier effect via PFIs for greater sensitivity and resolution. The research findings indicate that the proposed sensor has a good linear response to temperature and pressure testing at 16–400 °C, 0–1.0 MPa. It provides a pressure sensitivity of 114 nm/MPa and a temperature sensitivity of 176 pm/°C. Combining both ease of fabrication and unmodified optical fibers, this sensor can be a good candidate for mass production in the fields of gas sensing and environmental safety monitoring.

## 2. Fabrication and Operating Principle

### 2.1. Operating Principle

**Figure 1:**
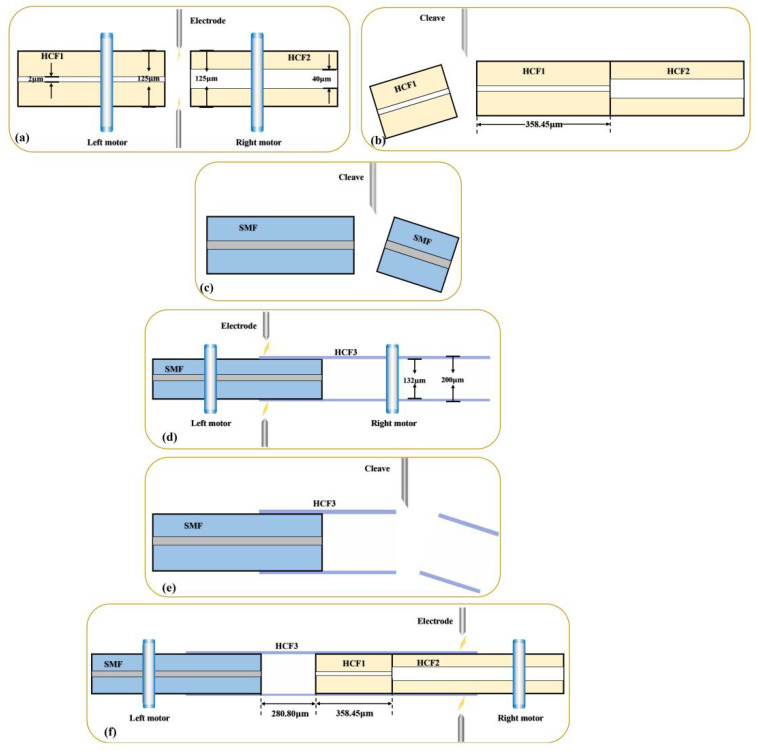
(**a**–**f**) Steps of the FPI sensor fabrication process.

The schematic diagram of the proposed harmonic Vernier FPI sensor is illustrated in Figure 1. First, a short segment of HCF1 (inner/outer diameter ~2 µm/125 µm) was spliced to the HCF2 (inner/outer diameter ~40 µm/125 µm) using a commercial fusion splicer (Fujikura Co., Ltd, Shanghai, China), as illustrated in Figure 1a; the purpose of this splicing is to ensure that the gas can access freely. After that, the length of HCF1 was reduced to 381.2 µm by cleaving the excessive part of the fiber using a mechanical cleaver (see Figure 1b). Next, a segment SMF with a flat-cleaved end (see Figure 1c) was inserted into the HCF3 (inner/outer diameter ~132 µm/200 µm), and the overlapping region between the SMF and HCF3 was fused using the fusion splicer (see Figure 1d). Following that, the HCF1 end of the HCF1-HCF2 fiber was flat-cleaved before it was inserted into the open end of the HCF3 segment to form an air cavity, which was sealed by splicing the overlapping region between HCF3 and HCF2 by the fusion splicer (see Figure 1f). HCF2 acts as the exchange channel between the cavity of HCF3 and the ambient air so that the air pressure inside the air cavity is always equivalent to the air pressure of the surrounding medium. Therefore, it is important to ensure the air cores of HCF1 and HCF2 remain intact during the arc-fusion splicing. A weaker fusion power and a shorter fusion time were chosen for the splicing to prevent the collapse of the air cores by the induced excessive heat.

### 2.2. Sensing Principle

**Figure 2:**
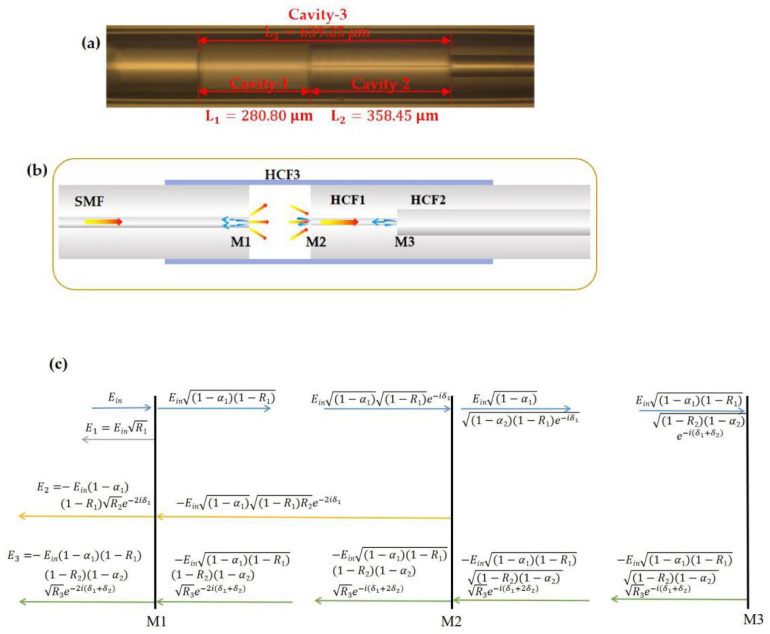
(**a**) The microscope image and (**b**) illustrative diagram of the FPI structure. (**c**) Three-reflector model.

Figure 2a shows the micrograph and dimension parameters of the manufactured FPI structure with three reflective interfaces—M1, M2, and M3 as illustrated in Figure 2b. Cavity–1 is basically an air cavity bounded between M1 and M2, while Cavity–2 is the hollow core of HCF1 bounded between M2 and M3. The measured lengths of Cavity–1 and Cavity–2 from the micrograph are L1 = 280.8 µm and L2 = 358.45 µm, respectively. It is worth noting that both HCF1 and HCF2 are of different hollow-core diameters with slightly different effective indices. The Fresnel reflection at M3 might be small, but it is not negligible. Therefore, there is another optical cavity (Cavity–3) bounded between the interfaces of M1 and M3 with an effective cavity length of L1 + L2 = 639.25 µm.

The reflection of the FPI is the result of multi-beam coherent superposition, and it can be well-described using a three-reflector model [29,30,31,32]. The relationship between the incident, reflected, and transmitted electric fields at each interface is depicted in Figure 2c. When light is emitted from the SMF to the sensor, reflections occur at M1, M2, and M3 interfaces. The corresponding electric fields *E*_1_, *E*_2,_ and *E*_3_ of the reflected beams can be expressed as follows:

(1) $$E_{1}=E_{in}\sqrt{R_{1}}$$

(2) $$E_{2}=E_{in}(1-\alpha_{1})(1-R_{1})\sqrt{R_{2}}$$

(3) $$E_{3}=E_{in}(1-\alpha_{1})(1-\alpha_{2})(1-R_{1})(1-R_{2})\sqrt{R_{3}}$$

where $R_{1}$, $R_{2}$ and $R_{3}$ are the corresponding reflectivities at M1, M2, and M3, respectively. $\alpha_{1}$ and $\alpha_{2}$ are the transmission losses at M1 and M2, respectively. The negative signs in Equations (2) and (3) represent the results of 180° phase shifts for the reflected beams that experience exterior reflection at M2 and M3. The resultant reflection intensity is as follows:

(4) $$\begin{array}{cc} Ir & =\left|E_{r}{}^{2}\right|={\left|E_{1}-E_{2}e^{\left(-2i\delta_{1}\right)}+E_{3}e^{\left(-2i\left(\delta_{1}+\delta_{2}\right)\right)}\right|}^{2}\\ & =E_{1}{}^{2}+E_{2}{}^{2}+E_{3}{}^{2}-2E_{1}E_{2}\cos(2\delta_{1})\\ & -2E_{2}E_{3}\cos(2\delta_{2})+2E_{1}E_{3}\cos(2\delta_{1}+2\delta_{2}) \end{array}$$

where $E_{in}$ is the incident electric field; $\delta_{1}=2\pi n_{1}L_{1}/\lambda$, $\delta_{2}=2\pi n_{2}L_{2}/\lambda$ are the single-trip propagation phase-shifts in Cavity–1 and Cavity–2, respectively. $n_{1}$ and $n_{2}$ are the effective indices of Cavity–1 and Cavity–2. $L_{1}$ and $L_{2}$ are the corresponding cavity lengths, and λ is the wavelength of the incident light.

The period of a conventional Vernier envelope can be expressed as follows:

(5) $$FSR_{envelope}=\frac{FSR_{1}\times FSR_{2}}{\left|FSR_{1}-FSR_{2}\right|}$$

where *FSR*_1_ and *FSR*_2_ are the corresponding *FSR* of Cavity–1 and Cavity–2.

(6) $$FSR_{1}=\frac{\lambda^{2}}{2n_{1}L_{1}}$$

(7) $$FSR_{2}=\frac{\lambda^{2}}{2n_{2}L_{2}}$$

The magnification factor (m-factor) of the conventional Vernier effect is associated with the ratio of the *FSR* of the Vernier envelope to the *FSR* of a single cavity interferometer:

(8) $$m=\frac{FSR_{envelope}}{FSR_{sensing}}=\frac{FSR_{1}{}_{}}{\left|FSR_{1}-FSR_{2}\right|}$$

Assuming that the optical path length of one of the cavities is i times that of the other cavity, which is $L_{2}=iL_{1}$ (*i* = order of harmonic), higher-order harmonics of the Vernier effect can be generated. The FSR of the upper envelope of the harmonic Vernier effect can be expressed as follows:

(9) $$FSR^{i}{}_{envelope}=\left|\frac{FSR_{1}\times FSR^{i}{}_{2}}{FSR_{1}-(i+1)FSR_{2}}\right|$$

It is noted that the upper Vernier envelope is independent of the order of the harmonics. The *FSR* of the internal envelope of the harmonic Vernier effect can be expressed as follows:

(10) $$FSR_{harmonic}^{i}=\left|\frac{(i+1)FSR_{1}^{}\times FSR_{2}^{i}}{FSR_{1}^{}-(i+1)FSR_{2}^{i}}\right|=(i+1)FSR^{i}{}_{envelope}$$

This indicates that the internal envelope *FSR* is i+1 times larger than the upper envelope *FSR*. Based on a similar mathematical approach in attaining Equation (6), the M factor of the harmonic Vernier effect can be expressed as follows:

(11) $$m_{harmonic}^{i}=\frac{FSR^{i}{}_{harmonic}}{FSR_{1}}=(i+1)m$$

Unlike the other reported Harmonic Vernier sensors, both cavities in the proposed optical are affected by the measured at the same time. None of them can serve as the reference for the measurement. One expects that the sensitivity of the Vernier envelope will depend on both interferometers. Therefore, the pressure sensitivity of the Vernier envelope can be expressed as follows:

(12) $$S_{envelope}=m_{1}S_{1}+m_{2}S_{2}$$

where *S*_1_ and *S*_2_ denote the sensitivities of two resonant cavities interferometers, respectively. *m*_1_ and *m*_2_ are the corresponding magnification factors. More explanation will be provided with the two selected resonant cavities in the next section. The same analysis applies to the temperature sensitivity of the Vernier envelope, leading to the same result. Since the interference frequencies of the two interferometers are slightly detuned, it causes one of the magnification factors of Equation (13) to turn negative. Consequently, it can be observed that the sensitivity of the envelope is determined by the difference between the sensitivities of each sensing interferometer, and the result is weighted by their respective magnification factors. Therefore, the sensitivity of the envelope can be calculated by taking into account the detuning between the interference frequencies of the two interferometers.

The magnification of the Vernier effect depends on the respective sensitivity of the two resonant cavities. It is believed that the pressure sensitivity of the harmonic effect comes from the contribution of Cavity–1 and Cavity–3. The pressure sensitivity of the harmonic Vernier envelope can be expressed as follows:

(13) $$S_{envelope}^{i}=\left(i+1\right)\left(m_{1}S_{1}+m_{2}S_{2}\right)$$

## 3. Experiments and Results Discussion 

**Figure 3:**
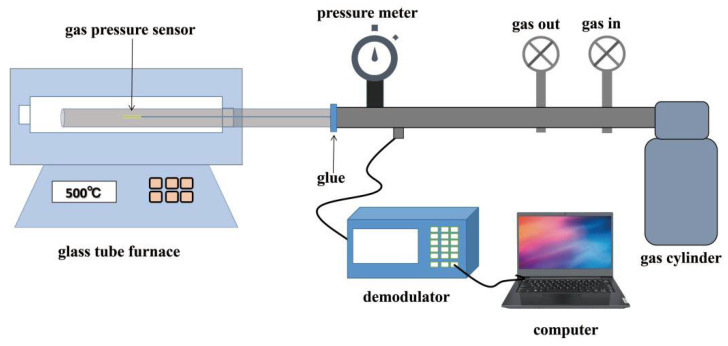
Experimental setup for the characterization of the sensor.

Figure 3 shows the experimental setup for the characterization of the sensor. It comprises a high-temperature pressure furnace, a gas cylinder (99.999% N2), an inlet and exhaust valve, a gas chamber, and a manometer (accuracy ± 0.001 MPa) that measures the pressure inside the gas chamber. The pressure in the gas chamber can be adjusted from 0.001 MPa to 1.0 MPa by the inlet and exhaust valve. Before the test, the sensor was positioned at the center of the hot zone in the tube furnace, and the tube was sealed to prevent gas leakage. During the test, the optical spectrum of the fiber sensor was analyzed using an FBG interrogator SM255 (Micron Optics Inc., Chamblee, GA, USA) with a wavelength resolution of ~1 pm, and the data were recorded using a laptop.

### 3.1. Spatial Frequency Spectral Analysis

**Figure 4:**
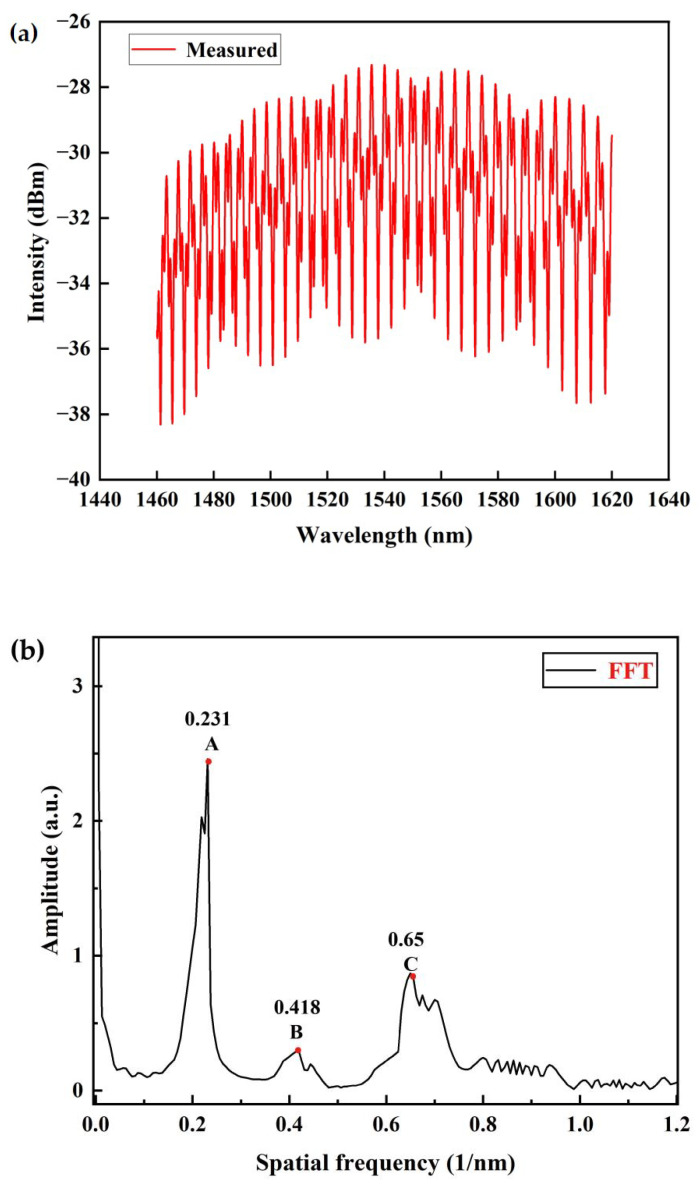
(**a**) The reflection spectrum of the device sample. (**b**) The corresponding spatial frequency spectrum.

The reflection spectrum within the wavelength range of 1460–1620 nm of the sensor under atmospheric pressure at room temperature is shown in Figure 4a. Figure 4b shows the fast Fourier transform (FFT) of the spectrum, in which the frequency peaks are marked as A, B, and C. The frequency peak, A = 0.231 nm^−1^ is associated with Cavity–1 (M1–M2) whereas the frequency peak, B = 0.418 nm^−1^ is associated with Cavity–2 (M2–M3). The frequency peak, C = 0.65 nm^−1^ is close to A + B = 0.649 nm^−1^, which suggests that it is associated with Cavity–3 (M1–M3), the combined cavity of Cavity–1 and Cavity–2 [33]. The broad frequency peaks of B and C are the results of the excitation of several higher-order modes in the HCF1. Based on the measured cavity lengths from Figure 2b, the spatial frequency of each cavity and combined cavity can be calculated using $\zeta =2nL/\lambda^{2}$, where n is the effective index of the cavity, and L is the cavity length. Table 1 summarizes the measured and calculated spatial frequencies for M1–M2, M2–M3, and M1–M3. The calculated spatial frequencies in the table were computed based on the measured cavity lengths, as stated in Figure 2b, and the assumed effective indices of 1.0 and 1.4 for cavities M1–M2 and M2–M3, respectively. The calculated spatial frequencies for peaks A and B are 0.234 nm^−1^ and 0.418 nm^−1^, which are close to the measured frequencies of 0.231 nm^−1^ and 0.418 nm^−1^. The spatial frequency for peak C is calculated to be 0.652 nm^−1^, which is the sum of the previous two calculated frequencies for peaks A and B. Both measured and calculated frequencies are in good agreement.

### 3.2. Pressure Measurement

**Figure 5:**
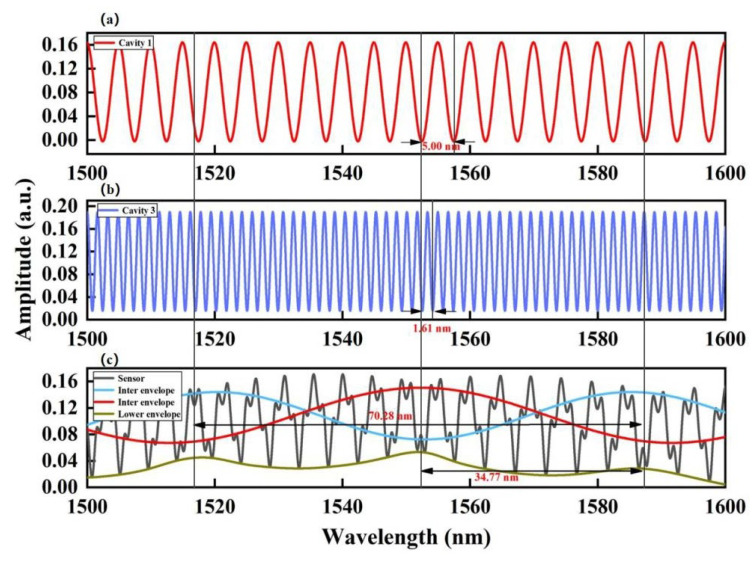
The corresponding interference spectrum for (**a**) Cavity–1 and (**b**) Cavity–3. (**c**) The measured optical spectrum of the sensor at room temperature and standard atmospheric pressure.

Based on the amplitude, peaks A and C are the only two dominating frequency components in the spectrum. Figure 4b shows the FFT results of the spectrum in Figure 4a, where corresponding frequency components at ~0.231 and 0.65 Hz are evident. In the spectral analysis, digital bandpass filters with bandpass ranges of 0.229–0.234 nm^−1^ and 0.647–0.652 nm^−1^ were applied to extract the individual interference spectra from peak A and peak C, respectively; the results are presented in Figure 5a,b. The *FSR*s of spectra in Figure 5a,b are estimated to be 5 nm and 1.51 nm, which are the reciprocals of the measured spatial frequencies 0.231 nm^−1^ and 0.65 nm^−1^. Figure 5c shows the original reflection spectrum of the sensor under the standard atmospheric pressure at room temperature. In the subsequent characterization of the sensitivity, the envelope within the chosen range of 1500–1600 nm is selected for the measurement. Both the red and blue curves represent the inner envelope curve of the original spectrum in Figure 5c.

In signal demodulation, the tracking of the *FSR* is not practical because it can be larger than the scanning range of the FBG interrogation system. On the other hand, the tracking of the intersection point of two envelopes is a more pragmatic approach for the harmonic Vernier sensor. The sensitivity of the harmonic Vernier envelope refers to the spectral shift of the intersection point with the pressure change and temperature change. The acquired FSRs of the inner envelope and lower-bound envelope are ${FSR}_{inner\_enve}$ = 70.28 nm and ${FSR}_{low\_enve}$ = 34.77 nm. Substituting them into Equation (13) yields

(14) $$S_{envelope}^{i}=(i+1)\frac{FSR_{low\_enve}}{FSR_{1}}\times S_{1}+\frac{FSR_{low\_enve}}{FSR_{2}}\times S_{2}$$

In the pressure characterization test, the proposed sensor was characterized in the pressure range of 0 MPa–1.0 MPa with an incremental step of 0.2 MPa under the same temperature conditions, starting from room temperature until 400 °C with an incremental step of 100 °C and repeating the above steps. A wait time of 5 min was applied at each pressure point to ensure the system had stabilized before the spectral data were recorded.

**Figure 6:**
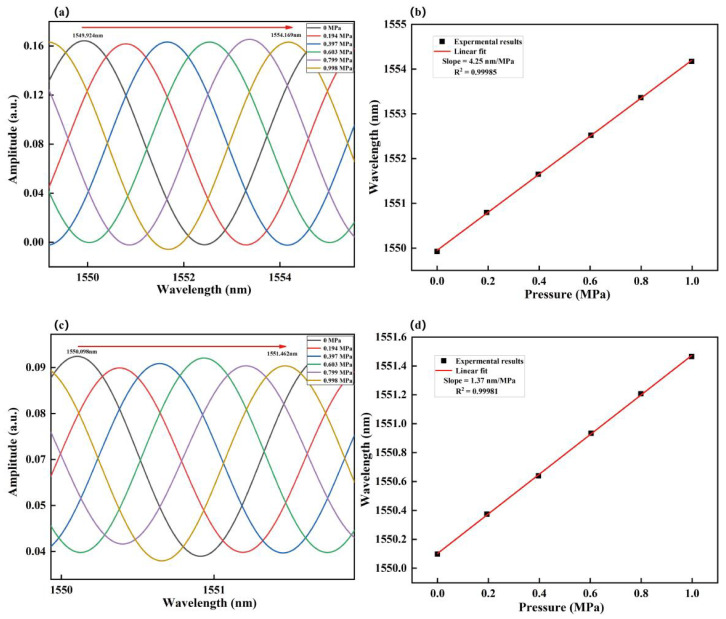
The extracted interference spectra of (**a**) Cavity–1 and (**c**) Cavity–3 at different gas pressures; The pressure responses of (**b**) Cavity–1 and (**d**) Cavity–3 at room temperature.

Figure 6a,c shows the pressure responses of the extracted interference spectra of Cavity–1 and Cavity–3 in the range of 0–1.0 MPa at room temperature. The peaks of the interference spectrum at ~1550 nm were employed as the indicators for the measurement. The pressure of the gas chamber was increased by injecting air into the gas chamber via an inlet valve, and the pressure was raised from 0 MPa to 1.00 MPa at an incremental step of 0.2 MPa. The equivalent variation of the air RI, $n_{air}$ is 1.00026–1.00048 RIU (incremental step of Δ*n* ~ 0.00003 RIU). The air pressure responses P = 0 MPa ($n_{air}$= 1.00026) and P = 1.00 MPa ($n_{air}$ = 1.00048) of Cavity–1 and Cavity–3 at room temperature are shown in Figure 6b,d. The resonance wavelengths of Cavity–1 and Cavity–3 redshift as the air pressure increases. The linear fitting results show that the air pressure sensitivities of Cavity–1 and Cavity–3 are 4.25 nm/MPa and 1.37 nm/MPa, respectively. Substituting 4.25 nm/MPa, 1.37 nm/MPa, 5 nm, and 1.61 nm into Equation (14), the calculated sensitivity of the envelope is 118.618 nm/MPa, and the measured sensitivity of the envelope is 114.356 nm/MPa, which deviates by less than 3%. Table 2 lists the relevant parameters, measurement sensitivity, and calculation sensitivity. Our measurement sensitivity agrees well with the theoretical calculation sensitivity.

**Figure 7:**
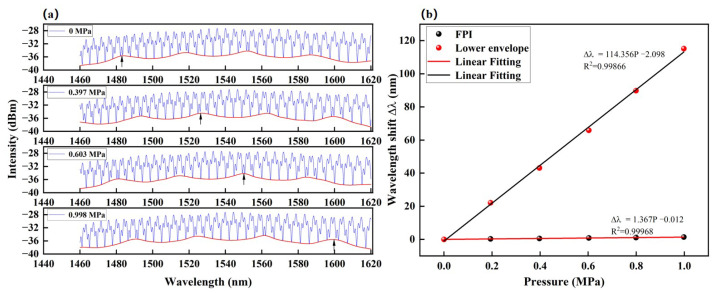
(**a**) The shift of the reflective spectra envelope versus gas pressure. (**b**) The pressure response of the Vernier envelope and FPI.

Figure 7a shows the spectral shift of the envelope with increasing gas pressure. The linear relationship between the Vernier envelopes and gas pressures is shown in Figure 7b. The measured pressure sensitivity of the Vernier envelope is 83 times greater than that of the low-frequency interference fringes (associated with the FPI of Cavity–1). The pressure-sensing capability of this sensor can be attributed to the fact that the air cavities inside the fiber structure are connected to the ambient air/pressure via the hollow core and air-lattice of HCF1, HCF2, and HCF3. This is the key to an instantaneous change in the air refractive index and optical path lengths of the cavities. Furthermore, no hysteresis effect was observed in the output response of the sensor during the process of increasing or decreasing the gas pressure. Since the detection of spectral shift is based on the dip of the Vernier envelope, the higher the frequency of the envelope fringe, the more precise the envelope dip position, and a larger detection range can be achieved. It can be seen that the envelope of the reflective spectra experienced a redshift of 114.2 nm at 1 MPa.

### 3.3. Temperature Measurement

**Figure 8:**
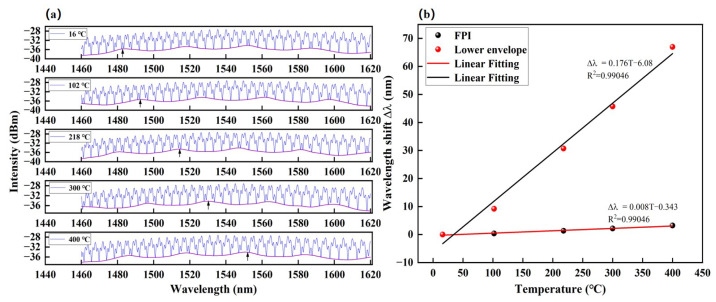
(**a**) The shift of the reflective spectra envelope versus temperature. (**b**) The linear fitting between the envelop wavelengths and different temperatures.

The temperature response of the sensor was also measured with the experimental setup described in Figure 3. In the thermal characterization test, the sensor was inserted and positioned at the center of the glass tube furnace, keeping the pressure constant to ensure that the test results are not affected by the pressure, and the temperature is adjusted. The annealing temperature was set to increase in steps from room temperature (25 °C) until 400 °C at the step size of 100 °C. The waiting time for each incremental step is 30 min to ensure a uniform temperature distribution inside the furnace, sufficient to stabilize the spectrum before recording it. Figure 8a shows the output spectra of the sensor at different temperatures. A total of 4 dips of the upper envelope and 3 peaks of the lower envelope can be observed in the wavelength range from 1460 nm to 1620 nm, respectively. It is worth noting that the amplitude of the lower envelope is larger than that of the upper envelope, so the spectral shift of the lower envelope can be employed as the sensing indicator.

The lower envelopes of the reflection spectra and the peak of the lower envelope exhibit clear red shifts with increasing temperature, as shown by the black arrow in Figure 8a,b, which illustrates the relationships between the envelope peak shifts and temperature. The linear fitting function of lower envelopes is ∆λ=0.176T−6.08, with good linear responses of ~0.99, respectively. The temperature sensitivity of the envelope was 176 pm/°C. This can be attributed to the thermo-optic effect and thermal expansion effect in the silica glass in Cavity–2, which is a part of Cavity–3. This explains the higher temperature sensitivity of high-frequency interference fringe (Cavity–3). On the other hand, the low-frequency interference spectra in Figure 8a are also analyzed. It is found that the low-frequency interference spectrum is almost insensitive to temperature change. This is because the low-frequency interference spectrum is associated with Cavity–1, which is mostly air that has no thermo-optic effect.

It can be found that with similar measurement ranges, the sensitivity of the gas pressure for a single FP cavity sensor is only 4.28 nm/MPa, which is less than 1/25 of the proposed sensor. Table 3 compares the performance of the proposed sensor with other related sensors. The proposed sensor has excellent performance in terms of pressure and temperature sensitivities.

## 4. Conclusions

In summary, a compact harmonic Vernier sensor has been experimentally demonstrated for gas pressure and temperature measurement. It is a three-reflector system that comprises three resonance cavities formulated by different fiber segments (HCF1, HCF2, HCF3, and SMF). By using a digital bandpass filter, the interference spectra of the desired harmonics can be extracted and analyzed separately. It was observed that the waveguide and material properties of the cavities have an impact on the temperature sensitivity. The experimental results show that the sensor has an excellent linear response to pressure response in the range of 0–1 MPa with a large operating temperature in the range of 16–400 °C. The measured pressure sensitivity is 114 nm/MPa, and the temperature sensitivity is 176 pm/°C. The corresponding detection limits are 8.77 Pa and 5.68 × 10^−3^ °C, respectively. Overall, the proposed sensor has shown great advantages in terms of robustness, simple fabrication, and high sensitivity. It has great potential in various applications, such as chemical processing, oil and gas, and aerospace industries.

## Figures and Tables

**Table 1:** The comparison between the measured and calculated spatial frequencies.

	Spatial Frequency (nm^−1^)		Effective Index, n	Measured Cavity Length, L (Figure 2b)	Cavity
Peak	Measurement (Figure 4b)	Calculation $\zeta =2nL/\lambda^{2}$			
A	0.231	0.234	1	280.80	Cavity–1 (M_1_–M_2_)
B	0.418	0.418	1.4	358.45	Cavity–2 (M_2_–M_3_)
C	0.65	0.652			Cavity–3 (M_1_–M_3_)

**Table 2:** The relevant parameters, measurement sensitivity, and calculation sensitivity.

$S_{1}$	$S_{2}$	${FSR}_{1}$	${FSR}_{2}$	${FSR}_{low\_enve}$	$S_{calculation}$	$S_{measurement}$
4.25 nm/MPa	1.37 nm/MPa	5 nm	1.61 nm	34.77 nm	118.618 nm/MPa	114.356 nm/MPa

**Table 3:** Comparison of gas pressure sensitivity and temperature sensitivity of different optical fiber sensors.

Sensor Structure	Measurement Range	Gas Pressure Sensitivity (nm/MPa)	Temperature Sensitivity (pm/°C)	Ref.
Diaphragm-free FPI	0.1–0.7 MPa 20–800 °C	4.28	14.8	[19]
Milled HCF + SMF	180–220 kPa	80.3	-	[34]
Parallel FPIs	0–0.7 MPa 25–600 °C	63.67	12.01	[35]
Parallel FPIs + Harmonics	0–200 KPa	279.52	-	[27]
HCF + Harmonics	0–100 KPa 30–100 °C	80.8	179.99	[26]
Cascade FPIs + Harmonics	0–1.0 MPa 16–400 °C	114	176	This work

## Data Availability

Not applicable.

## Abbreviations

The following are the abbreviations used in this article:

Abbreviations	Full Name
FPI	Fabry–Perot Interferometer
SMF	Single-Mode Fiber
FSR	Free Spectral Range
FP	Fabry–Perot
HCF	Hollow Core Fiber
FFT	Fast Fourier Transform
RI	Refraction Index
